# IL-12 Expands and Differentiates Human Vγ2Vδ2 T Effector Cells Producing Antimicrobial Cytokines and Inhibiting Intracellular Mycobacterial Growth

**DOI:** 10.3389/fimmu.2019.00913

**Published:** 2019-04-26

**Authors:** Rui Yang, Lan Yao, Ling Shen, Wei Sha, Robert L. Modlin, Hongbo Shen, Zheng W. Chen

**Affiliations:** ^1^Shanghai Key Lab of Tuberculosis, Clinic and Research Center of Tuberculosis, Shanghai Pulmonary Hospital, Institute for Advanced Study, Tongji University School of Medicine, Shanghai, China; ^2^Department of Microbiology and Immunology, Center for Primate Biomedical Research, University of Illinois College of Medicine, Chicago, IL, United States; ^3^Department of Microbiology, Immunology and Molecular Genetics, University of California, Los Angeles, Los Angeles, CA, United States; ^4^Division of Dermatology, David Geffen School of Medicine at University of California, Los Angeles, Los Angeles, CA, United States

**Keywords:** IL-12, Vγ2Vδ2 T cells, proliferation, differentiation, anti-tuberculosis

## Abstract

While IL-12 plays a key role in differentiation of protective CD4^+^ Th1 response, little is known about mechanisms whereby IL-12 differentiates other T-cell populations. Published studies suggest that predominant Vγ2Vδ2 T cells in humans/nonhuman primates (NHP) are a fast-acting T-cell subset, with capacities to rapidly expand and produce Th1 and cytotoxic cytokines in response to phosphoantigen (E)-4-hydroxy-3-methyl-but-2-enyl pyrophosphate (HMBPP) produced by *Mycobacterium tuberculosis* (Mtb) or others. However, whether IL-12 signaling pathway mediates fast-acting and Th1 or anti-microbial features of Vγ2Vδ2 T cells remains poorly defined. Here, we show that IL-12, but not other IL-12 family members IL-27/IL-35, apparently expanded HMBPP-activated Vγ2Vδ2 T cells. Although IL-12 and IL-2 similarly expanded HMBPP-activated Vγ2Vδ2 T-cell clones, the IL-12-induced expansion did not require endogenous IL-2 or IL-2 co-signaling during HMBPP + IL-12 co-treatment. IL-12-induced expansion of Vγ2Vδ2 T cells required the PI3K/AKT and STAT4 activation pathways and endogenous TNF-α signaling but did not involve p38/MAPK or IFN-γ signals. IL-12-expanded Vγ2Vδ2 T cells exhibited central/effector memory phenotypes and differentiated into polyfunctional effector cell subtypes which expressed TBX21/T-bet, antimicrobial cytokines IFN-γ, TNF-α, GM-CSF, and cytotoxic granule molecules. Furthermore, the IL-12-expanded Vγ2Vδ2 T cells inhibited the growth of intracellular mycobacteria in IFN-γ- or TNF-α-dependent fashion. Our findings support the concept that IL-12 drives early development of fast-acting Vγ2Vδ2 T effector cells in antimicrobial immune responses.

## Introduction

Interleukin-12 (IL-12) is a critical cytokine produced by monocytes/macrophages and dendritic cells in response to microbial pathogens (1–3). IL-12 also plays an important role in biological regulation of lymphocytes, as it can help to promote T cells for Th1 differentiation (4), augment proliferation of pre-activated T and NK cells (5), prompt the production of IFN-γ (6), and enhance cytolytic activity of cytotoxic T and NK cells (7). In fact, studies in humans and animals have demonstrated that IL-12 is essential for *in vivo* IFN-γ production and induction/maintenance of antigen-specific CD4^+^ Th1 cells for development of protective immunity against intracellular pathogens including resistance to *Mycobacterium tuberculosis* (Mtb) infection (8, 9). However, little is known about whether IL-12 can promote immune response or function of other T-cell populations that do not express CD4 during Mtb or other microbial infections.

γδ T cells appear to be a non-conventional T-cell population that contributes to both innate and adaptive immune responses against microbial infections (10). Vγ2Vδ2 T-cell subpopulation unique in humans and nonhuman primates (NHP) constitute 65–90% of total circulating human γδ T cells and remain the sole γδ T-cell subset capable of recognizing phosphoantigens such as the isopentenyl pyrophosphate (IPP) metabolite (11) and (E)-4-hydroxy-3-methyl-but-2-enyl pyrophosphate (HMBPP) produced by Mtb and other microbes (12). Studies in humans and NHP (13–17) have shown that IPP- or HMBPP-activated Vγ2Vδ2 T cells can readily produce Th1 cytokines IFN-γ/TNF-α and cytotoxic granule molecules perforin (PRF), granzyme A/B (GZMA/B), and granulysin (GNLY), and consistently exhibit antimicrobial and anti-cancer activities. On the other hand, activated Vγ2Vδ2 T cells can be expanded by IL-2, IL-7, IL-15, IL-21, IL-33, and Th17-related cytokines (13, 18–21). Furthermore, recent seminal studies in NHP models suggest that the phosphoantigen HMBPP-specific Vγ2Vδ2 T-cell subset can respond as fast-acting T cells, undergo rapid expansion and pulmonary trafficking and residence, and attenuate high-dose Mtb infection (10, 15, 16). However, whether IL-12 signaling pathway mediates fast-acting and Th1 or anti-microbial features of Vγ2Vδ2 T cells remains poorly defined (22, 23).

In the current study, we performed mechanistic experiments to test the hypothesis that IL-12, a key innate cytokine produced by Mtb infection of macrophages/DC, plays a role in the early development of fast-acting Vγ2Vδ2 T effector cells. Our study provides previously-unreported data implicating signaling pathways, cytokine networks and functional mechanisms whereby IL-12 expands and differentiates HMBPP-activated Vγ2Vδ2 T effector cells producing multiple anti-TB cytokines and inhibiting mycobacterial growth.

## Materials and Methods

### Expansion of Vγ2Vδ2 T Cells by HMBPP Plus Cytokines in PBMC Culture

The protocols for human blood samples for *in vitro* experimental procedures were evaluated and approved by the institutional review boards for human subjects' research and institutional biosafety committees at Shanghai Pulmonary Hospital. All subjects are adults and signed written informed consents. Human PBMC were isolated from collected fresh blood of healthy donors by density gradient centrifugation using Ficoll-Paque PLUS (GE) as described (16, 24). For expansion assay, 0.5 million PBMCs were cultured in the absence or presence of 10 ng/mL of HMBPP (provided by Dr. H. Jomaa, Germany), with/without 5 ng/mL IL-2 (R&D) or 25 ng/mL IL-12 (Miltenyi Biotech) at 200 ul in 96-U-well plate. Fresh culture media (RPMI1640 + 10% FBS, purchased from Life Technologies) with indicated cytokines was added into cultures every 2–3 day. CD4- or CD8- depleted PBMC were prepared from freshly PBMC by sorting CD4 or CD8 T cells out using MACS method (Miltenyi). In proliferation assays, CD4-depleted, CD8-depleted or undeleted PBMCs were labeled with 2 μM CFSE (Life Technology), washed out, then cultured with media, HMBPP, IL-12, or HMBPP + IL-12 for 7 days. Cells were harvested at day 7, and the proliferation of Vγ2Vδ2 T cells was analyzed by flow cytometry. In special assays, PBMCs were co-cultured with HMBPP + IL-12 or HMBPP + IL-2 with or without TNF-α (Invitrogen) or TGF-β1 (Peprotech) at indicated concentration. PBMCs were co-cultured with IL-2 or IL-12 stimulated by plate-coated 1 ug/ml anti-CD3 Ab (OKT3, BD) plus soluble 1 ug/ml anti-CD28 Ab (CD28.2, BD) or HMBPP for 7 days. The following neutralization antibodies and their corresponding isotype controls were used in antibody blocking assays: anti-IL-2 (MQ1-17H12; BD), IgG (R35-95, BD); anti-IL-2 (Polyclonal, AF219; R&D), IgG (Polyclonal, AB-108, R&D); anti-IFN-γ (MD-1, Biolegend), anti-TNF-α (MAb1, Biolegend) and IgG (MOPC-21, Biolegend). (+/–)-Lisofylline (LSF, ENZO) was used for IL-12/STAT4 axis-targeted inhibiting experiments. SB203580 and LY294002 purchased from Abmole, were used for inhibiting p38-MAPK and PI3K/AKT pathway, respectively. Inhibitors were used at indicated concentration and added along with cytokines every 2–3 days during 7-day-culture.

### Repertoire Analysis of Expanded Vγ2Vδ2 T Cells

Expanded Vγ2Vδ2 T cells from HMBPP + IL-12 or HMBPP + IL-2 cultures were used for RNA isolation and re-transcribed into cDNA library (25). Fragments containing Vγ2- and Vδ2-specific CDR3 sequences were amplified by standard PCR kit (Yeasen), using the following primers: 5′-ATCAACGCTGGCAGTCC-3′ and 5′-AAGGAAGAAAAATAGTGGGC-3′ for Vγ2 chain; 5′-GCAGGAGTCATGTCAGCCAT-3′ and 5′-GACAAGCGACATTTGTTCCA-3′ for Vδ2 chain (26). The PCR fragments were purified from PCR system by PCR products purification kit (Axygen), and then ligated into the pMD-19T cloning vector (Takara). The recombinant plasmids were transfected by heat shock into DH5α competent cells (Sanyou Biotech). Colonies were picked and grown overnight in 4 ml of Luria-Bertani broth containing Ampicillin (50 mg/ml). Plasmids were purified using the plasmid Miniprep kit (Axygen). The sequences of plasmids were determined by Huagen Biotech. CDR3 lengths and VDJ rearrangements were analyzed through IMGT/V-QUEST (27).

### Characterizing Memory Surrogates and Surface Markers on Expanded Vγ2Vδ2 T Cells

Cultured cells were washed with PBS, stained with Zombie Fixable Viability Kit (Biolegend), followed by staining with monoclonal Abs against special surface markers. For cell memory state analysis, cells were incubated with PB-anti-CD3 (SP34-2, BD), FITC-anti-Vγ2 (7A5, Thermo Scientific), PE-anti-Vδ2 (B6, Biolegend), BV785-anti-CD45RA (HI100, Biolegend), PE/Cy7-anti-CD27 (O323, Biolegend), PE/Cy5-anti-CD28 (CD28.2, Biolegend) for 20 min at room temperature in dark. For phenotyping of special surface markers, cells were incubated with PB-anti-CD3 (SP34-2, BD), FITC-anti-Vγ2 (7A5, Thermo Scientific), PE-anti-Vδ2 (B6, Biolegend), APC-anti-CCR5 (J418F1, Biolegend), PE/Cy5.5-anti-LFA-1 (TS1/18, Biolegend) for 20 min at room temperature in dark. Then cells were washed and fixed by fixing buffer (2% formalin in PBS), analyzed on an LSR Fortessa flow cytometer (BD).

### Intracellular Cytokine Staining (ICS) for Functional Evaluation

PBMCs cultured for 7 days were firstly treated with/without 40 ng/ml HMBPP, pulsing PE/CF594-anti-CD107a (H4A3, Biolegend), and BFA (GolgiPlug, BD) for 6 h. Then cells were stained with Zombie Fixable Viability Kit (Biolegend), incubated with PB-anti-CD3 (SP34-2, BD), FITC-anti-Vγ2 (7A5, Thermo Scientific), PE-anti-Vδ2 (B6, Biolegend) for 20 min at room temperature in dark. Cells were permeabilized for 30 min at 4 degrees (Cytofix/Cytoperm, BD). After wash, cells were incubated with BV711-anti-IFN-γ (4S.B3; Biolegend), PE/Cy7-anti-TNF-α (Mab11, Biolegend), Percp/Cy5.5-anti-GM-CSF (BVD2-21C11, Biolegend) for 30 min at room temperature in dark. Then cells were washed and analyzed on an LSR Fortessa flow cytometer (BD). CD107a was used to assess degranulation of cytotoxic molecules; IFN-γ, TNF-α, and GM-CSF was used to evaluating anti-mycobacteria effector function. Flow data were analyzed by FlowJo (TreeStar).

### Quantification of Gene Expression in Expanded Vγ2Vδ2 T Cells

RNA isolation from enriched and stimulated Vγ2Vδ2 T cells, reverse-transcription and PCR reactions were done as described in Qaqish et al. (16). Primers used for amplification were listed as following and synthesized from Sangon Biotech: *GNLY*-F, 5′-GTACTACGACCTGGCAAGAGCC-3′, *GNLY*-R, 5′-TCAGACAGGTCCTGTAGTCACG-3′; *PRF*-F 5′-ACTCACAGGCAGCCAACTTTGC-3′, *PRF*-R, 5′-CTCTTGAAGTCAGGGTGCAGCG-3′; *GZMA*-F 5′- CCACACGCGAAGGTGACCTTAA-3′, *GZMA*-R, 5′-CCTGCAACTTGGCACATGGTTC-3'; *GZMB*-F, 5′- CGACAGTACCATTGAGTTGTGCG-3′, *GZMB*-R, 5′-TTCGTCCATAGGAGACAATGCCC-3′; TBX21-F, 5′-ATTGCCGTGACTGCCTACCAGA-3', TBX21-R, 5′-GGAATTGACAGTTGGGTCCAGG-3′; Foxp3-F, 5′- GGCACAATGTCTCCTCCAGAGA-3',Foxp3-R, 5′-CAGATGAAGCCTTGGTCAGTGC−3′; *EF1A*-F, 5′-GATTACAGGGACATCTCAGGCTG-3′, *EF1A*-R, 5′-TATCTCTTCTGGCTGTAGGGTGG-3′. *EF1A* was used as a reference gene. Fold change was calculated with the delta C_t_ method.

### Intracellular Mycobacterial Growth Inhibition Assay

*Mycobacterium bovis* Bacillus Calmette-Guerin (BCG)-infected THP-1 and human monocytes-derived macrophages (hMDM) were prepared as target cells at MOI 10 as we previously described (24). Extracellular BCG were then washed out. To isolate or purify Vγ2Vδ2^+^ T cells, cells in HMBPP + IL-12 or HMBPP + IL-2 cultures were stained with PE-anti-Vδ2 (B6, Biolegend), and then incubated with anti-PE microbeads (Miltenyi Biotech) to minimize potential cross-linking activation. Vδ2^+^ T cells were then purified or enriched using MACS Separation columns (Miltenyi Biotech) according manufacturer's protocol, serving as effector cells. The purity of enriched population is 97 ± 2.2% for HMBPP + IL-12 co-cultures and 94 ± 2.06% for HMBPP + IL-2 co-cultures assessed by flow cytometry (Supplementary Figure 4C). Target cells (5 × 10^4^ cells/well) were cultured with media alone or with purified effector cells (5 × 10^5^ cells/well) at a ratio of E: T = 10: 1 in 96-well plates for 3 days. Autologous B cells, which were isolated or enriched by CD20 immunomagnetic microbeads (Miltenyi Biotech), were included in these co-culture systems and served as negative controls as described (15). In some special assays, 10 ug/ml of neutralization Abs against to IFN-γ (MD-1, Biolegend), TNF-α (MAb1, Biolegend), and IgG (MOPC-21, Biolegend) were used to block T cell effector functions (16). Mycobacteria viability were quantified via counting CFU as previously described (24).

### Statistical Analysis

Statistical analyses of data were performed using *t*-test or ANOVA methods with Prism 6.0 (GraphPad).

## Results

### IL-12 Family Cytokines Differently Regulate the Expansion of HMBPP-Specific Vγ2Vδ2 T Cells

**Figure 1:**
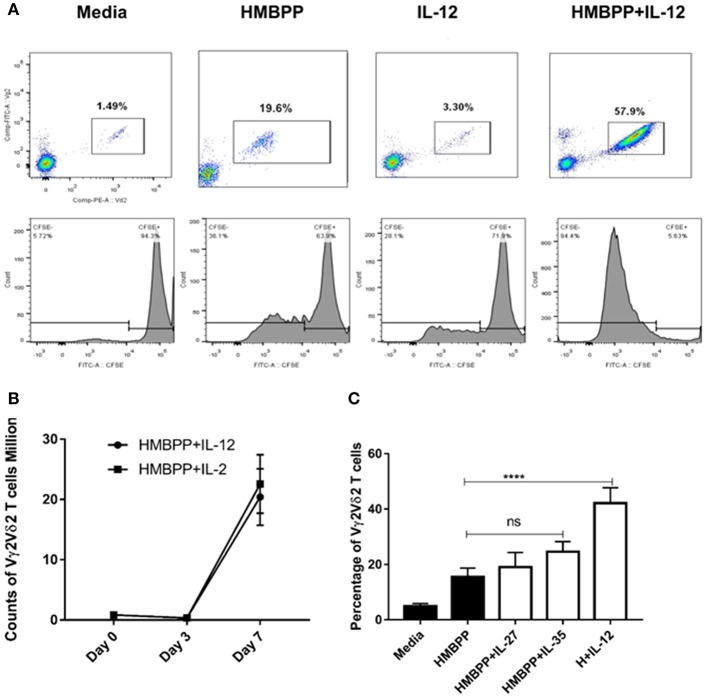
IL-12, but not IL-27 or IL-35, significantly drives the proliferation and expansion of HMBPP-activated Vγ2Vδ2 T cells. **(A)** Representative flow cytometric plots shown cellular population augmentation (upper panels) and cellular division (lower panels) of Vγ2Vδ2 T cells after 7-day *ex vivo* co-culture with media, HMBPP (10 ng/ml), IL-12 (25 ng/ml), or the combination of HMBPP + IL-12. Cells gated on Live cells, lymphocytes, single not doublet/triplet, and then CD3^+^ T cells. Vγ2Vδ2 T cells were expressed as percentage in total CD3^+^ T cells. Cellular division or proliferation was determined by the percentage of diluted/lower CFSE fluorescence intensity of Vγ2Vδ2 T cells. **(B)** Kinetics of absolute number of Vγ2Vδ2 T cells during expansion treated by HMBPP + IL-12 or HMBPP + IL-2. Data shown as mean ± SEM of three independent experiments pooled from 12 healthy controls. **(C)** Both IL-27 and IL-35 failed to induce the expansion of Vγ2Vδ2 T cells in PBMC followed by HMBPP stimulation. PBMCs from healthy controls were treated by HMBPP (10 ng/ml) with or without either IL-27 or IL-35 (25 ng/ml) for 7 days. Percentages of Vγ2Vδ2 T cells in PBMC were determined by flow cytometry. The bar plot shown the percentages of Vγ2Vδ2 T cells in CD3^+^ T cells were similar in HMBPP + IL-27 or IL-35 vs. HMBPP only cultures. Data shown as mean ± SEM of five independent experiments pooled from 25 healthy controls. NS, not significant (p > 0.05) for statistics. ^****^p < 0.0001, when HMBPP + IL-12 group is compared to media group (ANOVA, Dunnett's test). The specific bioactivity of recombinant IL-12 cytokine was validated in our previous publication (24) in the blockade assay using the anti-IL-12 neutralizing mAb, and this anti-IL-12 neutralizing mAb can significantly reduce HMBPP + IL-12 expansion (not shown).

Published studies suggest that phosphoantigen HMBPP-specific Vγ2Vδ2 T-cell subset represents fast-acting innate-like T cells, undergoing rapid expansion and pulmonary trafficking and residence during Mtb infection (10, 16, 28). We wondered whether IL-12, a key innate cytokine produced by initial Mtb infection of macrophages/DC (2, 3), plays a role in early expansion of HMBPP-activated Vγ2Vδ2 T cells. To address this, we examined whether IL-12 could trigger the proliferation and expansion of Vγ2Vδ2 T cells that were stimulated with the phosphoantigen HMBPP. The source and validation of recombinant human IL-12 were reported in our previous study (24). While HMBPP or IL-12 treatment alone did not induce expansion, the combined HMBPP and IL-12 treatment (HMBPP + IL-12) significantly induced robust proliferation or cell division as shown by CFSE staining (Figure 1A lower panel). Consistently, HMBPP + IL-12 acted like HMBPP + IL-2 to induce increases in percentage numbers of Vγ2Vδ2 T cells after 7 days of culture (Figures 1A,B).

Given that IL-12 and IL-23 are members of IL-12 cytokine family (2, 3), and IL-23 also expands HMBPP-activated Vγ2Vδ2 T cells (16, 19), we examined whether the other two IL-12-family cytokines, IL-27 and IL-35, could have similar effects on the proliferation or expansion of HMBPP-activated Vγ2Vδ2 T cells. Surprisingly, both IL-27 and IL-35 failed to expand HMBPP-activated Vγ2Vδ2 T cells as compared to HMBPP treatment alone (Figure 1C). Together these results demonstrated that like IL-23, IL-12 promoted expansion of HMBPP-stimulated Vγ2Vδ2 T cells. These data also suggest that cytokines in the IL-12 family mediate surprisingly diverse functional effects on Vγ2Vδ2 T cells.

### IL-12 and IL-2 Similarly Expand HMBPP-Activated Vγ2Vδ2 T-Cell Clones, but the IL-12-Induced Expansion Does Not Require Endogenous IL-2 or IL-2 Signaling

**Figure 2:**
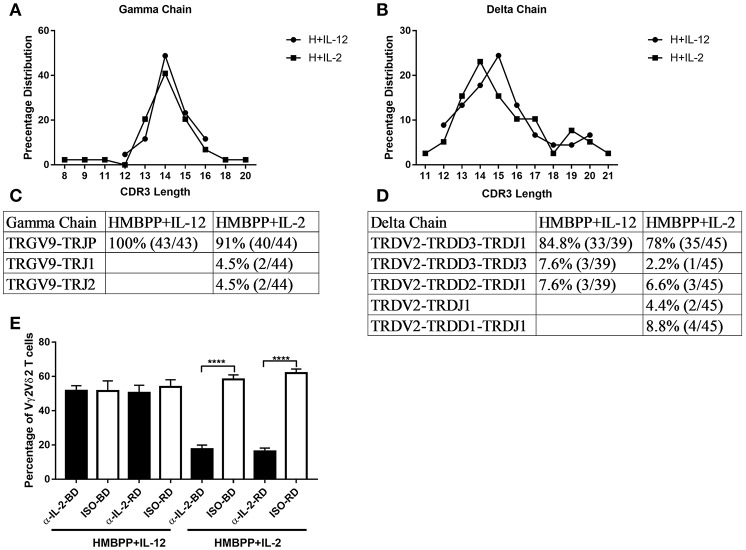
IL-12 and IL-2 similarly expand predominant clones of HMBPP-activated Vγ2Vδ2 T cells, but the IL-12-induced expansion does not require endogenous IL-2. **(A–D)** A comparison of the percentage of CDR3 length **(A,B)** and analysis of VDJ sequences **(C,D)** in Vγ2- and Vδ2-bearing TCR expressed by cells expanded by HMBPP + IL-12 and HMBPP + IL-2, respectively. RT-PCR was used to specifically amplify the CDR3 regions of Vγ2- and Vδ2-bearing TCR cDNA. **(E)** Endogenous IL-2 is not involved in IL-12 induced expansion of HMBPP-activated Vγ2Vδ2 T cells. The bar graph shows that anti-IL-2 neutralizing mAbs from two sources (αIL-2-BD and αIL-2-RD) fail to block the ability of IL-12 to expand HMBPP-activated Vγ2Vδ2 T cells (left panel), but these anti-IL-2 mAbs not isotype controls (ISO-BD/ISO-RD) significantly reduced the IL-2 expansion of HMBPP-activated Vγ2Vδ2 T cells in the HMBPP + IL-2 culture. PBMC were co-cultured with HMBPP (10 ng/ml) plus IL-2 (5 ng/ml) or IL-12 (25 ng/ml) in the presence or absence of 5 ug/ml anti-IL-2/IL-12 antibody or isotype controls for 7 days. Data shown as mean ± SEM of four independent experiments pooled from 20 healthy controls, ^****^p < 0.0001, *t*-test.

Since IL-2 can strikingly expand HMBPP-activated Vγ2Vδ2 T cells *in vivo* (29), we compared the IL-2 and IL-12 signals for the expansion of HMBPP-activated γδ T cells. Here we demonstrated that IL-12 shared with IL-2 the ability to expand Vγ2Vδ2 T cells in the presence of HMBPP phosphoantigen, but not anti-CD3 + anti-CD28 treatment (Supplementary Figure 1). We then employed PCR-based cloning and sequencing to analyze clonotypic TCR V(D)J sequences and frequencies of expanded Vγ2Vδ2 T cells as we described earlier (25). The proof-of-concept experiment was aimed to compare IL-12 and IL-2 expansion modes at clone levels rather than frequency of clones. Here, we found that IL-12 and IL-2 similarly expand predominant clones of HMBPP-activated Vγ2Vδ2 T cells expressing the identical VDJ junctional nucleotide sequences and CDR3 lengths (Figures 2A–D). This result suggests that while HMBPP phosphoantigen predominantly activates selected Vγ2Vδ2 T-cell clones, these clones can similarly be expanded by IL-12 and IL-2 signals. Next, we investigated whether IL-12-induced expansion of activated Vγ2Vδ2 T cells requires co-signaling from endogenous IL-2 produced by HMBPP + IL-12 co-culture. Our experiments indicated that IL-12-induced expansion did not require endogenous IL-2 or IL-2 signaling activation during HMBPP + IL-12 co-treatment, as anti-IL-2 neutralizing antibodies from two sources failed to affect the magnitude of the IL-12 expansion of HMBPP-activated Vγ2Vδ2 T cells (Figure 2E). In contrast, each of these anti-IL-2 neutralizing antibodies clearly abrogated the ability of IL-2 to expand HMBPP-activated Vγ2Vδ2 T cells (Figure 2E). Together, these results suggest that although IL-12 and IL-2 similarly expand HMBPP-activated Vγ2Vδ2 T cell clones, the IL-12-induced expansion does not require endogenous IL-2 or IL-2 co-signaling. Furthermore, we also found CD4 and CD8 T cells are not required for IL-12-induced proliferation of HMBPP-activated Vγ2Vδ2 T cells (Supplementary Figure 2). Data also implicate that IL-12 alone without HMBPP does not efficiently induce Vγ2Vδ2 T cell expansion even in the presence of the anti-CD3 + anti-CD28 stimulation.

### The IL-12-Induced Expansion of HMBPP-Activated Vγ2Vδ2 T Cells Requires Endogenous TNF-α, but Not IFN-γ

**Figure 3:**
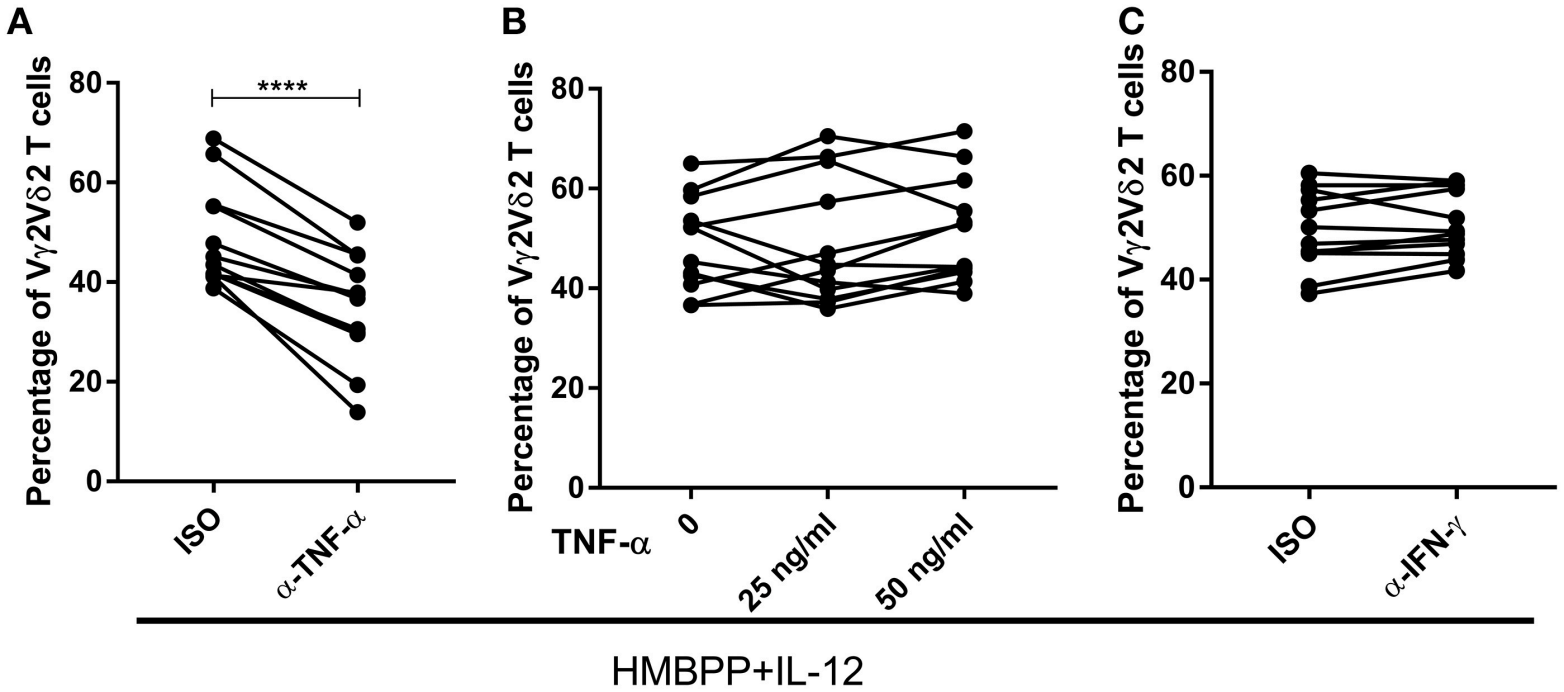
The IL-12-induced expansion of HMBPP-activated Vγ2Vδ2 T cells requires endogenous TNF-α, but not IFN-γ. Pooled graph data of the percentage of Vγ2Vδ2 T cells in CD3^+^ T cells expanded after the 7-day in the PBMC culture treated with HMBPP + IL-12 in the presence or absence of 10 ug/ml cytokine-neutralizing antibodies of anti-IFN-γ **(A)** or anti-TNF-α mAb **(C)**. Data are mean ± SEM of three independent experiments pooled from 12 healthy controls ^****^p < 0.0001, paired *t*-test. **(B)** Exogenous TNF-α did not enhance the ability of IL-12 to expand HMBPP-activated Vγ2Vδ2 T cells. TNF-α (25 or 50 ng/ml) was added to HMBPP + IL-12 cultures, and cells were cultured for 7 days prior to measuring expansion of Vγ2Vδ2 T cells. Data shown as mean ± SEM of three independent experiments pooled from 12 healthy controls.

TNF-α, but not IFN-γ, has been reported to be a positive regulator in the IL-2 induced expansion of IPP-activated Vγ2Vδ2 T cells (30). On the other hand, our previous studies showed that endogenous IFN-γ is critical for the IL-23-induced expansion of HMBPP-activated Vγ2Vδ2 T cells (19, 31). Consistent with previous data (16, 30, 32), we demonstrated that HMBPP + IL-12 and HMBPP + IL-2 expansion of Vγ2Vδ2 T cells led to massive production of TNF-α in the culture supernatant, suggesting the origin of this cytokine (Supplementary Figure 3). We therefore sought to determine whether endogenous TNF-α and IFN-γ after HMBPP + IL-12 co-activation were required in IL-12-induced expansion of HMBPP-activated Vγ2Vδ2 T cells. Interestingly, blocking the endogenous TNF-α signal using neutralizing mAbs significantly reduced or abrogated the ability of IL-12 to expand HMBPP-activated Vγ2Vδ2 T cells (Figure 3A). However, the addition of exogenous TNF-α did not significantly alter HMBPP + IL-12-mediated expansion of Vγ2Vδ2 T cells (Figure 3B). This was consistent with published data suggesting that TNF-α itself does not expand Vγ2Vδ2 T cells (30). Concurrently, we found that the IFN-γ blockade by neutralizing mAb did not reduce or abrogate the ability of IL-12 to expand HMBPP-activated Vγ2Vδ2 T cells (Figure 3C). Thus, these data demonstrated that TNF-α signaling was required for optimal cell proliferation of Vγ2Vδ2 T cells.

### Both PI3K/AKT and STAT4 Pathways Are Involved in the IL-12-Induced Expansion of HMBPP-Activated Vγ2Vδ2 T Cells

**Figure 4:**
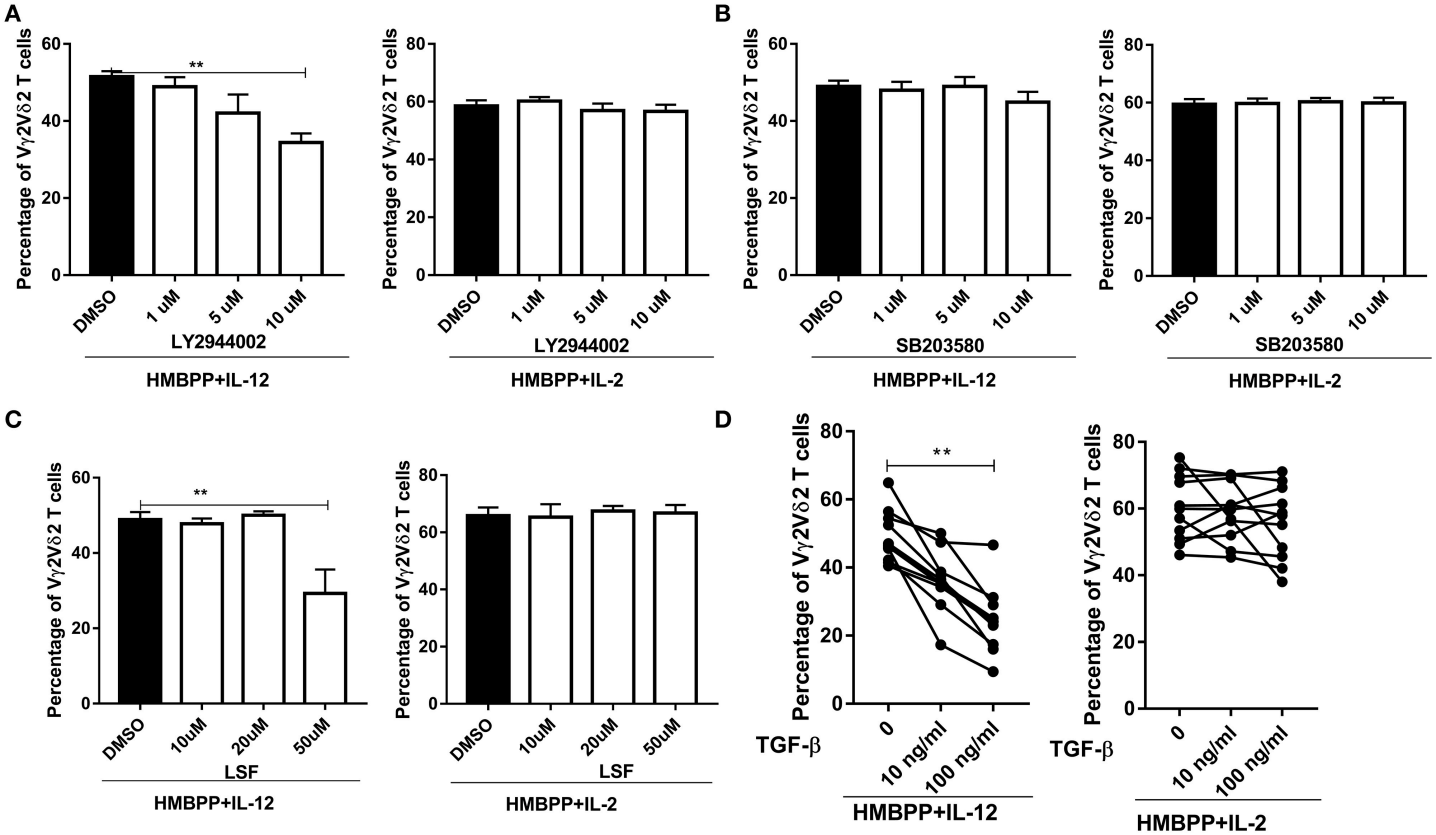
Both PI3K/AKT and STAT4 pathways, but not p38/MAPK, are involved in the IL-12-induced expansion of HMBPP-activated Vγ2Vδ2 T cells. **(A–C)** are graph data showing that PI3K/AKT and STAT4, but not P38/MAPK pathway, were required for HMBPP + IL-12 expansion of Vγ2Vδ2 T cells was reduced by the chemical blockers for PI3K/AKT and STAT4 pathways, but not those for P38/MAPK. PBMCs were co-cultured for 7 days with HMBPP + IL-2 or HMBPP + IL-12 in the presence of escalating doses (1, 5, 10 μM) of LY2944002 (inhibitor for PI3K/AKT), SB203580 (inhibitor for p38/MAPK), or doses (10, 20, 30, 40, 50 μM) of LSF (inhibitor for STAT4) or DMSO. Data are mean ± SEM of three independent experiments pooled from 12 healthy controls. ^**^p < 0.01, vs. media group (ANOVA, Dunnett's test). **(D)** Exogenous TGF-β significantly reduce the ability of IL-12, but not IL-2, to expand HMBPP-activated Vγ2Vδ2 T cells. TGF-β (10 or 100 ng/ml) was added to the PBMC cultures treated with HMBPP + IL-12 or HMBPP + IL-2 (n = 12), and cells were cultured for 7 days prior to the flow-based analysis of the expansion of Vγ2Vδ2 T cells. Each dot represents one healthy control. ^**^p < 0.01 vs. control group (paired *t*-test).

It has been shown that binding of IL-12 to the IL-12R triggers activation of the PI3K/ATK, p38-MAPK, and JAK1/TYk2-STAT4 pathways (2, 3). This raised an interesting question as to which of these selected signaling pathways contributes to IL-12-induced expansion of HMBPP-activated Vγ2Vδ2 T cells. To address this, we employed three well-documented small molecular inhibitors: SB203580 (inhibitor for p38/MAPK), LY2944002 (inhibitor for PI3K/AKT), and LSF (inhibitor for STAT4), respectively, in the cultures treated with HMBPP + IL-12 or controls. We found that LY294002, but not SB20358, significantly reduced or inhibited the ability of IL-12 to expand HMBPP-activated Vγ2Vδ2 T cells in a dose-dependent manner (Figures 4A,B). Moreover, the addition of LSF at 50 μM impaired the ability of IL-12, but not IL-2, to expand HMBPP-activated Vγ2Vδ2 T cells (Figure 4C). These results further support a model in which IL-12 employs different pathways from IL-2 for the growth and expansion of HMBPP-activated Vγ2Vδ2 T cells.

Transforming growth factor-β1 (TGF-β) has been reported to interfere with IL-12 signaling via impairing STAT4 activation and inhibiting IL-12-induced T-cell proliferation (33). However, an inhibitory effect of TGF-β on the IL-12 signaling activation of HMBPP-specific Vγ2Vδ2 T cells has not been addressed. Here, we show that the addition of exogenous TGF-β significantly reduced or inhibited the ability of IL-12, but not IL-2, to expand HMBPP-activated Vγ2Vδ2 T cells (Figure 4D).

Together, these results suggest that IL-12 activates the PI3K/AKT and STAT4 signaling pathways to induce the expansion of HMBPP-activated Vγ2Vδ2 T cells.

### Vγ2Vδ2 T Cells Expanded by HMBPP + IL-12 Exhibit Central/Effector Memory Phenotypes and Express Tissue Trafficking Markers

**Figure 5:**
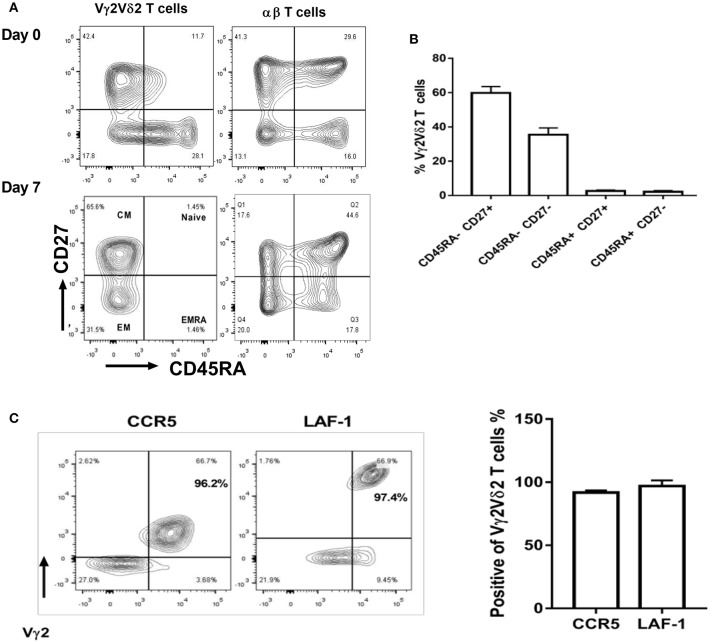
Vγ2Vδ2 T cells expanded by HMBPP + IL-12 exhibit central/effector memory phenotypes and maintain tissue trafficking markers. **(A)** Shown in the left are representative flow-cytometry quadrats displaying memory surrogate markers of Vγ2Vδ2 T cells based on CD27 and CD45RA expression in HMBPP + IL-12 cultures at day 0 and 7. Data at day 0 are similar to those in the medium only control culture. Shown in the right are representative flow cytometry histograms uncovering the phenotype of the αβ T cells that are cultured and gated in cytometry similarly to Vγ2Vδ2 T cells. **(B)** Bar graph shows that Vγ2Vδ2 T cells expanded by HMBPP + IL-12 express effector and central memory phenotypes. **(C)** Flow histogram and graph data show that expanded Vγ2^+^Vδ2^+^ T cells maintain expression of tissue-trafficking/residence markers CCR5 and LAF-1. Flowcytometry data are gated on live individual lymphocytes, CD3^+^, then Vγ2Vδ2^+^, and then representative markers. Data shown are mean ± SEM of three independent experiments pooled from 15 healthy controls.

Next, we sought to characterize the immune phenotypes of Vγ2Vδ2 T cells expanded by HMBPP + IL-12 using the cell-surface markers recently reported (34). Compared to day 0, the majority of Vγ2Vδ2 T cells expanded at day 7 by HMBPP + IL-12 exhibited central memory (CD45RA-CD27^+^) and effector memory (CD45RA-CD27-) phenotypes, but not naïve (CD45RA^+^CD27^+^) or terminally differentiated (CD45RA^+^CD27-) markers (Figures 5A,B).

We then investigated whether Vγ2Vδ2 T cells expanded *in vitro* by HMBPP + IL-12 expressed lung-tissue homing surrogate markers like those γδ T cells increased *in vivo* by the IL-2 + HMBPP administration (16). We found that most of these Vγ2Vδ2 T cells expanded by HMBPP + IL-12 indeed maintained the tissue-homing marker CCR5 (29) as well as LFA-1, a surrogate for T cell-APC interactions in tissues (Figure 5C). Taken together, these results suggest that the Vγ2Vδ2 T cells expanded by HMBPP + IL-12 exhibit the phenotypic potentials relevant to robust proliferation and tissue trafficking.

### Vγ2Vδ2 T Cells Expanded by HMBPP + IL-12 Differentiate Into Polyfunctional Effector Cells Expressing Antimicrobial IFN-γ, TNF-α, GM-CSF and Tri-CTL Molecules

**Figure 6:**
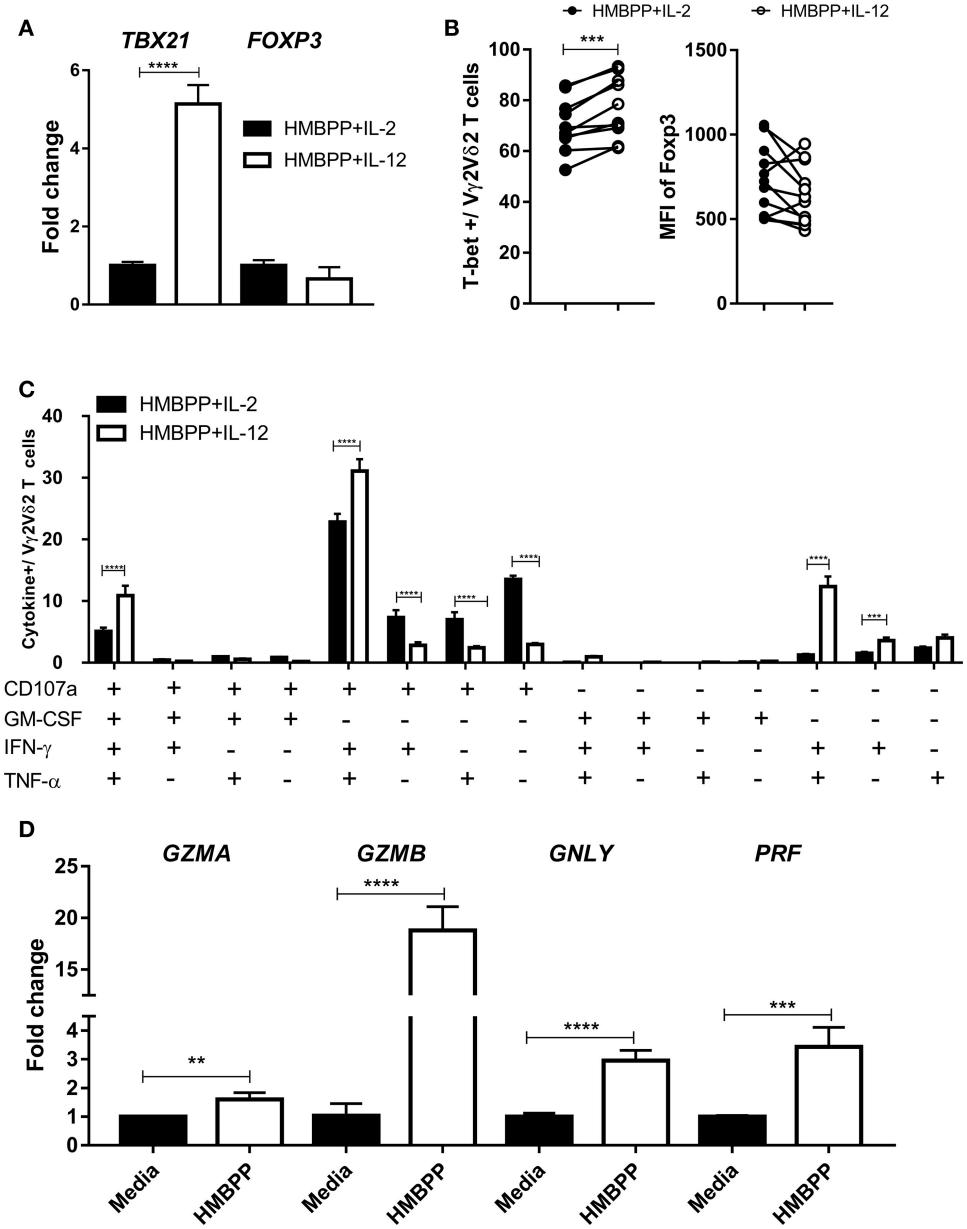
Vγ2Vδ2 T cells expanded by HMBPP + IL-12 differentiate into polyfunctional effector cells expressing antimicrobial IFN-γ, TNF-α, GM-CSF, and tri-CTL cytokines. **(A)** Vγ2Vδ2 T cells expanded by HMBPP + IL-12 expressed higher level of TBX21 than those expanded by HMBPP/IL-2. PBMC were cultured with HMBPP + IL-12 (white bars) or HMBPP + IL-2 (black bars) for 7 days, and expanded Vγ2Vδ2 T cells were purified. Then, enriched Vγ2Vδ2 T cells were used for RNA isolation and determination of the expression levels of TBX21 and FOXP3. Cells from HMBPP + IL-2 treatment served as control setting. Shown are mean ± SEM of three independent experiments pooled from 15 healthy controls, ^****^p < 0.0001, *t*-test. **(B)** Pooled flow-cytometry data (12 healthy controls) show the frequencies of T-bet^+^ cells (left) and mean fluorescence index (MFI) expression of Foxp3 (right) in gated Vγ2Vδ2 T cells expanded by HMBPP + IL-12 or HMBPP + IL-2. Each dot represents one healthy control. ^***^p < 0.001, paired *t*-test. **(C)** Percentage numbers of Vγ2Vδ2 T cells expanded by HMBPP + IL-12 or HMBPP + IL-2 could produce CD107a, IFN-γ, TNF-α, and GM-CSF in 15 combinations in response to HMBPP stimulation. Using Boolean analysis, the bar graph shows percentages of individual multi-functional effector subsets for Vγ2Vδ2 T cells expanded by HMBPP + IL-12 (white bars) and HMBPP + IL-2 (black bars). Gating was on individual live lymphocytes, CD3^+^ T, then Vγ2^+^Vδ2^+^ T cells and then those cytokine markers. Shown are mean ± SEM of three independent experiments pooled from 15 healthy controls, ^****^p < 0.0001, ^***^p < 0.001, *t*-test. **(D)** Bar graph shows fold changes in expression levels of GZMA, GZMB, GNLY, and PRF in HMBPP + IL-12-expanded Vγ2Vδ2 T cells after HMBPP or media stimulation. Data are from four independent experiments pooled from 15 healthy controls. ^****^p < 0.0001, ^***^p < 0.001, ^**^p < 0.01, *t*-test. Representative flow cytometric histograms are in Supplementary Figure 5A.

It has been well documented that IL-12 helps to differentiate antigen-stimulated CD4^+^ T cells into effector Th1 cells, characterized by expression of the transcription factor T-bet and production of IFN-γ (2, 3). Here, we compared T-bet or Foxp3 expression on HMBPP-activated Vγ2Vδ2 T cells following IL-12 or IL-2 treatment. The expression level of T-bet mRNA was significantly higher in Vγ2Vδ2 T cells expanded by HMBPP + IL-12 than those by HMBPP + IL-2, as revealed by RT-qPCR (Figure 6A). Consistently, Vγ2Vδ2 T cells expanded by HMBPP + IL-12 displayed a significantly greater MFI of fluorescence-stained T-bet protein than those γδ T cells activated by HMBPP + IL-2 (Figure 6B; Supplementary Figure 4A). However, there was no significant difference in Foxp3 mRNA and protein between the two groups (Figures 6A,B; Supplementary Figure 4B).

Next, we examined the HMBPP/IL-12-driven effector functions according to the production of specific cytokines. Vγ2Vδ2 T cells expanded by HMBPP + IL-12 were assessed for the ability to produce anti-microbial cytokines using intracellular cytokine staining (ICS) after HMBPP re-stimulation. We focused on IFN-γ, TNF-α, GM-CSF, or CD107a, as they were all necessary for anti-TB effector function or CTL function (14, 16). The results for production and co-production of these cytokines are summarized in 15 individual and combinational displays in Figure 6C and Supplementary Figures 5A–B. Overall, co-culture with HMBPP + IL-12 resulted in higher frequencies of Vγ2Vδ2 T effector cells producing IFN-γ or TNF-α than did HMBPP + IL-2 (Figure 6C; Supplementary Figure 5). Notably, Vγ2Vδ2 T cells expanded by HMBPP + IL-12 displayed a higher frequency of poly-effector functional T cells co-producing IFN-γ and TNF-α and CD107a or these 3 plus GM-CSF than did those by HMBPP + IL-2(Figure 6C; Supplementary Figure 5). These polyfunctional Vγ2Vδ2 T effector cells have been implicated as a surrogate marker for a protective anti-TB immune response (31).

It has recently been shown that CD8^+^ tri-cytotoxic T cells producing Granzyme B, granulysin, and perforin (Tri-CTL) exhibit a stronger ability to kill intracellular Mtb than those CTL producing only one or two of these three cytokines (35). We therefore assessed Vγ2Vδ2 T cells expanded by HMBPP + IL-12 for the expression of these CTL granule molecules which contribute to killing of intracellular pathogens (14–16, 36). We found that HMBPP re-stimulation significantly up-regulated expression of the tri-CTL molecules as well as GZMA (Figure 6D).

### Vγ2Vδ2 T Cells Expanded by HMBPP + IL-12 Inhibit the Growth of Intracellular Mycobacteria in an IFN-γ- or TNF-α-Dependent Fashion

**Figure 7:**
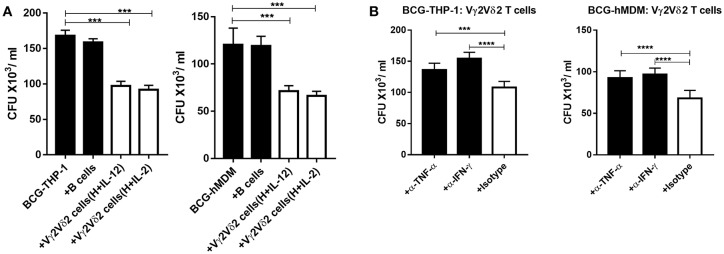
Vγ2Vδ2 T cells expanded by HMBPP + IL-12 inhibit intracellular mycobacteria growth in IFN-γ- and TNF-α-dependent fashions. **(A)** HMBPP + IL-12-expanded Vγ2Vδ2 T cells inhibit intracellular BCG growth in THP-1 and hMDM cells, respectively. Vγ2Vδ2 T cells enriched from HMBPP + IL-12 or HMBB + IL-2 co-cultures by positive selection (see Supplementary Figure 1D for the purity) were co-cultured with BCG-infected THP-1 (left panel) or hMDM (right panel) cells at an E: T ratio = 10:1 for 72 h. The reduction of CFU counts induced by Vγ2Vδ2 T cells was significantly more striking than that by B cells or media only. **(B)** Blocking assays using neutralizing mAb show that Vγ2Vδ2 T cells enriched from HMBPP + IL-12 cultures require effector molecules, IFN-γ and TNF-α, to inhibit intracellular mycobacteria. Vγ2Vδ2 T cells enriched from HMBPP + IL-12 cultures were incubated with BCG-infected THP-1 and hMDM cells at an E: T ratio of 10: 1 for 3 days in the presence/absence of 10 ug/ml neutralizing antibodies against IFN-γ, TNF-α, or matched Isotype. Data are mean ± SEM of three independent experiments pooled from 15 healthy controls, ^****^p < 0.0001, ^***^p < 0.001 vs. control (ANOVA, Dunnett's test).

Given that Vγ2Vδ2 T cells expanded by HMBPP + IL-12 expressed multiple anti-mycobacterial cytokines, we tested a possibility that these expanded cells could directly restrict intracellular mycobacteria growth. We performed a proof-of-concept experiment using BCG-infected targets, as Vγ2Vδ2 T effector cells capable of restricting intracellular BCG replication can also similarly inhibit intracellular Mtb growth (14–16). Since our earlier studies showed that activated Vγ2Vδ2 T effector cells more strikingly inhibited Mtb growth than resting γδ T cells (15, 16), here we comparatively evaluated IL-12- and IL-2-activated Vγ2Vδ2 T effector cells for BCG growth inhibition. Thus, expanded Vγ2Vδ2 T effector cells were co-cultured with BCG-infected THP-1 or autologous human monocyte-derived macrophages (hMDM) and then assessed for their ability to influence intracellular BCG growth. We found that Vγ2Vδ2 T effector cells purified from HMBPP + IL-12 cultures (Supplementary Figure 4C) significantly inhibited BCG growth in THP-1 and autologous hMDM target cells, compared to the controls autologous B cells or media alone (Figure 7A). These results were consistent with the previous observation that Vγ2Vδ2 T effector cells generated *in vivo* during infection or phosphantigen + IL-2 administration could limit intracellular Mtb or *Listeria monocytogenes* growth (15, 16, 37, 38). Finally, we performed mechanistic experiments to examine whether endogenous IFN-γ or TNF-α contributed to the ability of HMBPP + IL12-expanded Vγ2Vδ2 T cells to inhibit intracellular mycobacterial growth. Interestingly, both anti-IFN-γ and anti-TNF-α neutralizing mAbs significantly reduced or abrogated the ability of expanded Vγ2Vδ2 T cells to inhibit BCG growth in THP-1 and hMDM cells (Figure 7B). Thus, these results support the view that Vγ2Vδ2 T cells expanded by HMBPP + IL-12 inhibit intracellular mycobacteria growth in an IFN-γ- and TNF-α-dependent fashions.

## Discussion

To our knowledge, the current study provides new evidence that IL-12 helps to promote expansion and differentiation of HMBPP-activated Vγ2Vδ2 T cells through signaling activities involving PI3K/AKT *and* STAT4 and TNF-α pathways, but not p38/MAPK, IL-2, IFN-γ networks. Vγ2Vδ2 T cells expanded by HMBPP + IL-12 display a memory phenotype of rapid proliferation, produce/co-produce anti-microbial cytokines IFN-γ, TNF-α, GM-CSF, and CD107a, express the Mtb-killing tri-CTL cytotoxic granule molecules GZMB, GNLY, and PRF. Importantly, these expanded Vγ2Vδ2 T cells can inhibit intracellular BCG growth in autologous hMDM and THP-1 cells. These findings support the hypothesis that IL-12, a key innate cytokine produced by initial Mtb infection, helps to drive early development of fast-acting Vγ2Vδ2 T effector cells in anti-TB immune responses (10, 15, 16, 28).

The IL-12 effects on Vγ2Vδ2 T cells have been evaluated *in vitro* in humans with HIV-1 infection (32, 39) and cancer (40). IL-12 helps to produce IFN-γ/TNF-α in the responder subset of HIV-1-infected humans, but fails to induce the activation of Vγ2Vδ2 T cells from anergic HIV-infected persons (32). While IL-12 and IL-18 synergize Vγ2Vδ2 T cell-mediated cytotoxicity against tumor cells (23), IL-18 enhances the proliferative and recall response of Vγ2Vδ2 T cells from HIV-1-infected individuals (39). Here, we extend the published studies to demonstrate that the defined IL-12 signaling expands and differentiates human Vγ2Vδ2 T effector cells producing antimicrobial cytokines and inhibiting intracellular mycobacterial growth.

IL-12 family cytokines include IL-12, IL-23, IL-27, and IL-35 and have specific roles in the initiation, expansion, and control of immune responses of αβ CD4^+^ T cells in Mtb infection (41). Our current and earlier studies demonstrate that only IL-12 and IL-23 in the IL-12 family can induce robust proliferation/expansion and functional differentiation of HMBPP-activated Vγ2Vδ2 T cells (16, 19). It is noteworthy that absence or mutation of IL-12p40 or the IL-12Rβ1 (the shared component for the IL-12 and IL-23 receptor) correlate well with the incidence of TB (42, 43). The genetic defects in humans may impact potential anti-TB immunity involving both CD4^+^ Th1/Th17 and major Vγ2Vδ2 T-cell subsets. IL-12 and IL-23 promote protective anti-TB CD4^+^ Th1 and Th17 cells, respectively (44), and expand and differentiate HMBPP-specific Vγ2Vδ2 T cells. HMBPP-specific Vγ2Vδ2 T cells exist only in humans and NHP, constitute 65–90% of total circulating human γδ T cells. Recent NHP studies using *in vivo* HMBPP + IL-2 expansion of Vγ2Vδ2 T cells and adoptive transfer of Vγ2Vδ2 T cells demonstrate that Vγ2Vδ2 T effector cells can protect against high-dose Mtb infection (15, 16).

Our data implicate signaling requirements or functional mechanisms for the IL-12-induced expansion/differentiation of HMBPP-activated Vγ2Vδ2 T cells. The IL-12 expansion of Vγ2Vδ2 T cells requires signaling via STAT4 and PI3K/AKT, but not p38/MAPK. This is in line with the published observation that PI3K/AKT pathway is required for IL-12-induced Th1 maturation of CD4^+^ T cells (45). The data of IL-12 signaling requirements strongly suggest that IL-12 directly expands HMBPP-activated Vγ2Vδ2 T cells, rather than an indirect consequence. This notion is also supported by the fact that IL-12 acts like IL-2 to selectively expand Vγ2Vδ2 T cells activated by HMBPP, but not by anti-CD3^+^anti-CD28 stimulation. Nevertheless, the IL-12-STAT4 and PI3K/AKT pathways are quite different from the IL-2 signaling pathway or mechanisms (20, 21). Consistently, IL-12-induced expansion of HMBPP-activated Vγ2Vδ2 T cells is independent upon endogenous IL-2 or IL-2 signaling, although IL-2 and IL-12 share the ability to expand predominant clones of HMBPP-selected γδ T cells. This is also different from the IL-23-mediated expansion of Vγ2Vδ2 T cells, which involves endogenous IL-2 (16). Interestingly, endogenous TNF-α signaling is involved in both IL-12 and IL-2 signaling expansion of HMBPP-activated Vγ2Vδ2 T cells. These findings suggest that TNF-α signaling contributes to IL-2 and IL-12 expansion of multiple anti-TB T effector subsets including CD4^+^Th1, CD8^+^CTL, the predominant Vγ2Vδ2 T subset and NKT cells. These TNF-α cytokine networks help explain further the observation that anti-TNFα mAb treatment in RA patients with LTBI leads to dysfunction of CD8^+^ T cells or other T effectors, with consequence of reactivation of tuberculosis (46).

The current study also identifies the antagonistic cytokine negatively regulating the IL-12-induced expansion of HMBPP-activated Vγ2Vδ2 T cells. Specifically, we find that TGF-β can negatively regulate the HMBPP + IL-12 expansion of Vγ2Vδ2 T cells. Virtually, TGF-β has been found to significantly inhibit the IL-12–induced phosphorylation of the STAT4, leading to a decrease in IL-12–induced STAT4 binding to DNA and subsequent inactivation of αβ T cells (33). The increased production and activity of TGF-β in TB patients (47) may help to explain dysfunction of TB-specific Vγ2Vδ2 T effector cells (31).

Vγ2Vδ2 T effector cells expanded by HMBPP + IL-12 exhibit central/effector memory and tissue trafficking phenotypes of robust proliferation potential. ~60% of these Vγ2Vδ2 T cells express the CD45RA-CD27^+^ central memory phenotype linked to cytokine production (48), and ~40% of them displayed the effector memory phenotype, which are depleted in active TB and TB/HIV-1 coinfection (49). Our recent adoptive transfer study demonstrates that Vγ2Vδ2 T effector cells with such phenotypes can rapidly traffic to and accumulate in pulmonary compartment and protect against high-dose Mtb infection in NHP (16). Similar phenotypes and anti-TB immunity are also seen during cHMBPP + IL-2 administration as immune manipulation of Mtb-infected macaques (15).

Our data show that HMBPP + IL-12 coactivation differentiates and enables Vγ2Vδ2 T cells to acquire the pleiotropic capability to produce multiple anti-TB cytokines that can inhibit or kill intracellular Mtb bacilli (14–16). Particularly, Vγ2Vδ2 T cells expanded by HMBPP + IL-12 can produce and co-produce IFN-γ and TNF-α and GM-CSF. In this context, these γδ T effector cells express Mtb-killing tri-CTL granule molecules GZMB, GNLY and PRF. It is important to note that IFN-γ and TNF-α and GM-CSF are well-defined cytokines capable of inhibiting Mtb growth and that CD8^+^ tri-CTL expressing GZMB, GNLY, and PRF are the CTL subset that can kill intracellular Mtb more efficiently than others (35).

Consistent with the anti-TB cytokine profiles, Vγ2Vδ2 T cells expanded by HMBPP + IL-12 can efficiently inhibit intracellular BCG growth in human THP-1 macrophages and autologous hMDM. The mechanism by which Vγ2Vδ2 T cells expanded by HMBPP + IL-12 inhibit intracellular BCG growth appears to involve IFN-γ and TNF-α as shown by cytokine-blocking experiments. This is consistent with previous data indicating that TNF-α and IFN-γ, as well as PRF, GNLY, and granzyme A inhibit intracellular Mtb growth (14–17, 36). Although the current study does not test the inhibition of Mtb growth, earlier studies demonstrate that Vγ2Vδ2 T effector cells capable of restricting intracellular BCG replication can also similarly inhibit intracellular Mtb growth (14, 16). It is also noteworthy that we focused on IL-12- expanded Vγ2Vδ2 T effector cells, as resting un-activated Vγ2Vδ2 T cells would not efficiently produce those anti-TB cytokines or potently inhibit mycobacterial growth (14, 16).

Thus, the current study provides new information demonstrating that IL-12 augments the proliferation and expansion of HMBPP-activated Vγ2Vδ2 T cells. Data implicate cytokine signaling networks in which IL-12 enables Vγ2Vδ2 T cells to differentiate to polyfunctional effector cells producing multiple anti-TB cytokines and inhibiting mycobacterial growth. Findings support the hypothesis that IL-12, a key innate cytokine produced by initial Mtb infection, may help to drive early development of fast-acting Vγ2Vδ2 T cells in anti-TB immune responses (10, 16, 28). Given that IL-12 and IL-2 similarly expand HMBPP-activated Vγ2Vδ2 T-cell clones but act via distinct mechanisms (20, 21), targetting these two powerful cytokines may provide a strategy to enhance antimicrobial Vγ2Vδ2 T cells responses to intervene in patients with multi-drug resistant and/or disseminated tuberculosis.

## Ethics Statement

The protocols for human blood samples for *in vitro* experimental procedures were evaluated and approved by the institutional review boards for human subjects' research and institutional biosafety committees at Shanghai Pulmonary Hospital.

## Author Contributions

HS, RY, and ZC designed the study. RY and LY performed the experiments, data collection, and analysis. LS, HS, and ZC initiated the project, critically discussed the data. ZC and HS obtained the findings. RY, HS, WS, and ZC wrote and revised the manuscript. All authors provided the approval of the manuscript for submission.

### Conflict of Interest Statement

The authors declare that the research was conducted in the absence of any commercial or financial relationships that could be construed as a potential conflict of interest.
