# Corrigendum: IL-12 Expands and Differentiates Human Vγ2Vδ2 T Effector Cells Producing Antimicrobial Cytokines and Inhibiting Intracellular Mycobacterial Growth

**DOI:** 10.3389/fimmu.2019.01742

**Published:** 2019-07-26

**Authors:** Rui Yang, Lan Yao, Ling Shen, Wei Sha, Robert L. Modlin, Hongbo Shen, Zheng W. Chen

**Affiliations:** ^1^Shanghai Key Lab of Tuberculosis, Clinic and Research Center of Tuberculosis, Shanghai Pulmonary Hospital, Institute for Advanced Study, Tongji University School of Medicine, Shanghai, China; ^2^Department of Microbiology and Immunology, Center for Primate Biomedical Research, University of Illinois College of Medicine, Chicago, IL, United States; ^3^Department of Microbiology, Immunology and Molecular Genetics, University of California, Los Angeles, Los Angeles, CA, United States; ^4^Division of Dermatology, David Geffen School of Medicine at University of California, Los Angeles, Los Angeles, CA, United States

**Keywords:** IL-12, Vγ2Vδ2 T cells, proliferation, differentiation, anti-tuberculosis

There is an error in the **Funding** statement. The correct Name for the “Chinese National Major Projects” is the “National Program Project.”

There are also errors in the grant numbers in **Funding** statement. The correct numbers for the “National Program Project” are “Grants 2018ZX10731301-006-001 (HS/SPH), 2013ZX10003009-002 (HS/IPS).” The correct number for the “Clinical Research Plan of SHDC” is “16CR1028B (WS/SPH),” and the correct numbers for the National Institutes of Health R01 grants are “OD015092/RR13601, HL64560, and HL129887 (ZC).”

A correction has therefore been made to the **Funding** statement:

“This work was supported by the National Program Project Grants 2018ZX10731301-006-001 (HS/SPH), 2013ZX10003009-002 (HS/IPS), Clinical Research Plan of SHDC 16CR1028B (WS/SPH), and the National Institutes of Health R01 grants OD015092/RR13601, HL64560, and HL129887 (ZC).”

The authors apologize for these errors and state that they do not change the scientific conclusions of the article in any way. The original article has been updated.

