# Supplementary material for: IL-12 Expands and Differentiates Human Vγ2Vδ2 T Effector Cells Producing Antimicrobial Cytokines and Inhibiting Intracellular Mycobacterial Growth
Source: Front Immunol. 2019 Apr 26;10:913. doi: 10.3389/fimmu.2019.00913 (PMC6497761; doi:10.3389/fimmu.2019.00913)
Supplement: Supplementary file 1 [file Data_Sheet_1.docx]

Supplementary Materials

# IL-12 expands and differentiates human Vγ2Vδ2 T effector cells producing antimicrobial cytokines and inhibiting intracellular mycobacterial growth

Rui Yang^1^, Lan Yao^1^, Ling Shen^2^, Wei Sha^1, *^, Robert L. Modlin^3,4^, Hongbo Shen^1, *^, and Zheng W. Chen^2^

^1^Clinic and Research Center of Tuberculosis, Shanghai Key Lab of Tuberculosis, Shanghai Pulmonary Hospital, Institute for advanced study, Tongji University School of Medicine, Shanghai 200433, China.

^2^Department of Microbiology and Immunology, Center for Primate Biomedical Research, University of Illinois College of Medicine, Chicago, IL 60612.

^3^Department of Microbiology, Immunology and Molecular Genetics, University of California, Los Angeles, Los Angeles, CA 90095.

^4^Division of Dermatology, David Geffen School of Medicine at University of California, Los Angeles, Los Angeles, CA 90095.

*Corresponding author: Dr. Hongbo Shen and Dr. Wei Sha, Room 209, Building 12, 507 Zhengmin Road, Shanghai 200433, China. E-mail address: [hongboshen109@hotmail.com](mailto:hongboshen109@hotmail.com); [shfksw@126.com](mailto:shfksw@126.com).


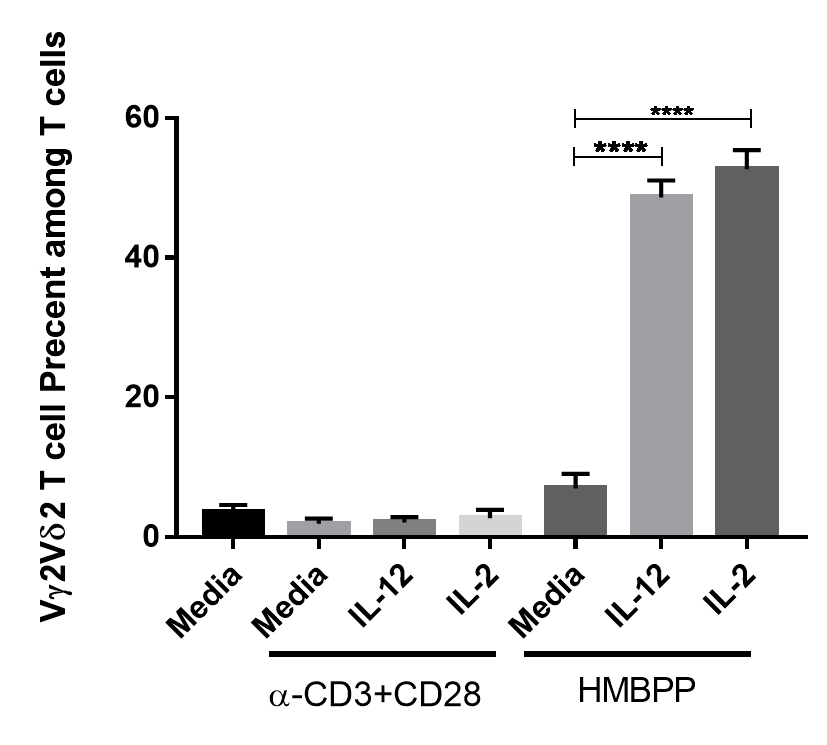


### Suppl Fig.1 IL-12 shared with IL-2 the ability to expand Vγ2Vδ2 T cells in the presence of HMBPP phosphoantigen, but not anti-CD3/anti-CD28 antibody

Bar graph showing percentages of Vγ2Vδ2 T cells among T cells in indicated cultures. PBMC (n=6) were cultured with media, anti-CD28+anti-CD3 Abs plus IL-12 or IL-2 as well as HMBPP plus IL-12 or IL-2 for 7 days. Data are presented as mean ± SEM. **** *p* < 0.0001 versus Media group (ANOVA, Dunnett’s test). Cell culture conditions were described in the Methods section in the text.


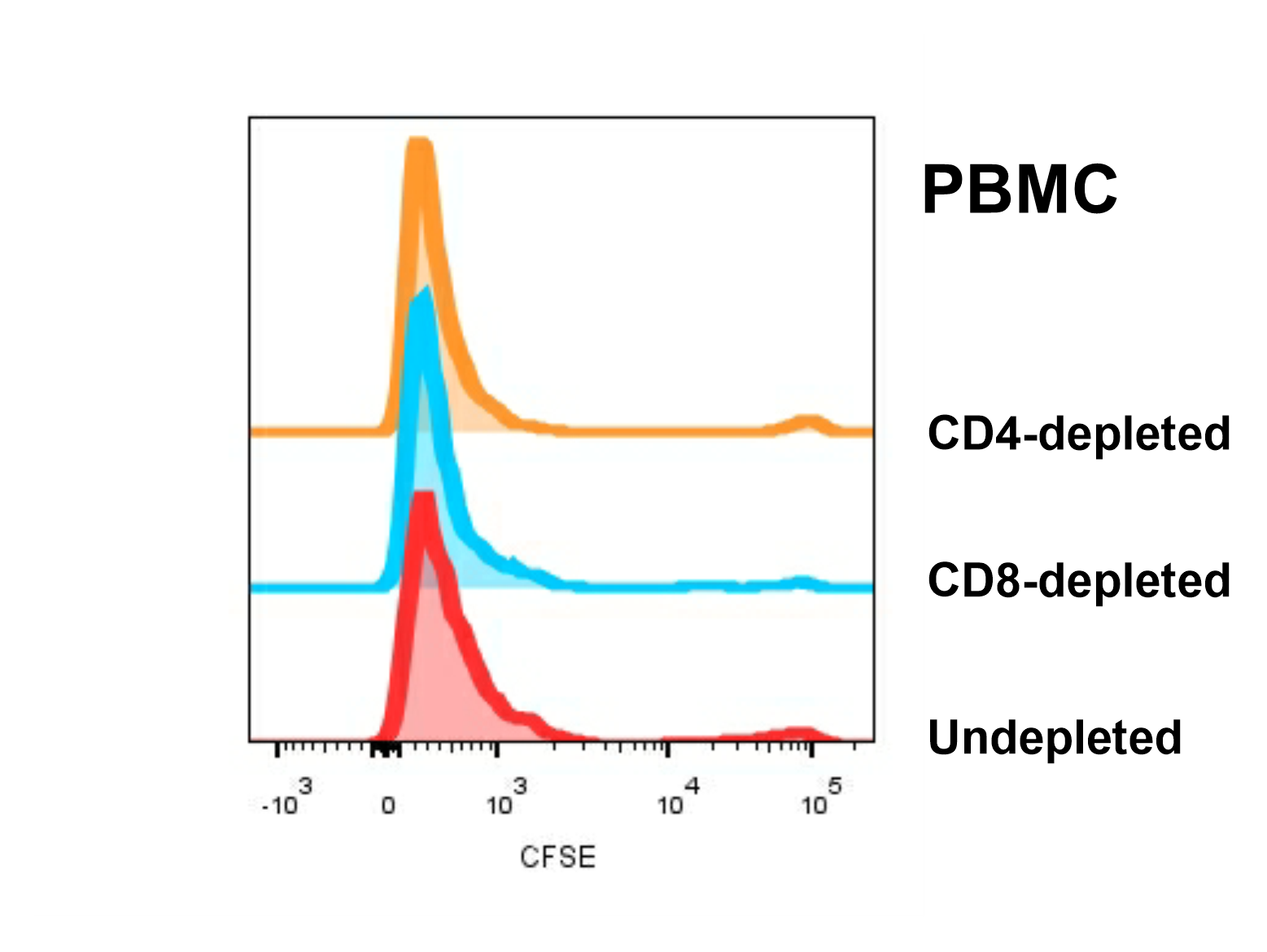


### Suppl Fig.2 IL-12–induced expansion of HMBPP-activated Vγ2Vδ2 T cells is not dependent on CD4 or CD8 T cells.

Representative CFSE dilutions of expanded Vγ2Vδ2 T cells in cultures of CD4-, CD8- and un-depleted PBMCs treated by HMBPP+IL-12 for 7 days.


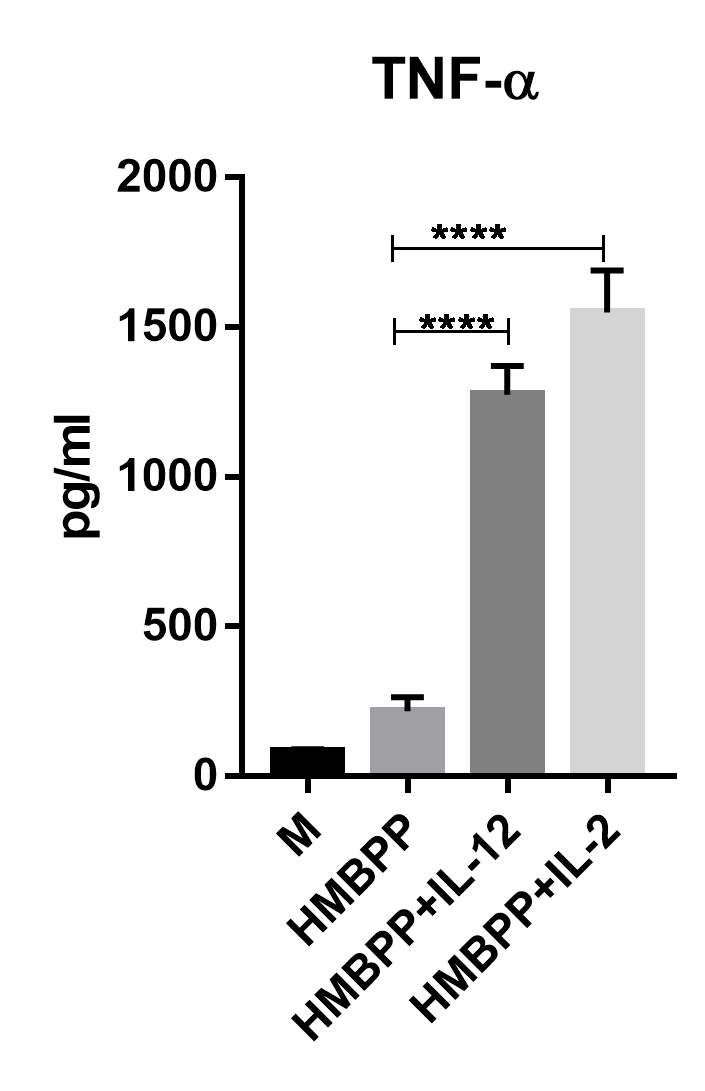


### Suppl Fig.3 Both HMBPP+IL-12 and HMBPP+IL-2 co-stimulation induce the production of TNF-α during expansion of Vγ2Vδ2 T cells.

Bar graph showing the concentration of TNF-α in the supernatants from various cultures indicated in Fig.1A. Data are presented as mean ± SEM pooled from 12 healthy controls. **** *p* < 0.0001 versus Media group (ANOVA, Dunnett’s test).


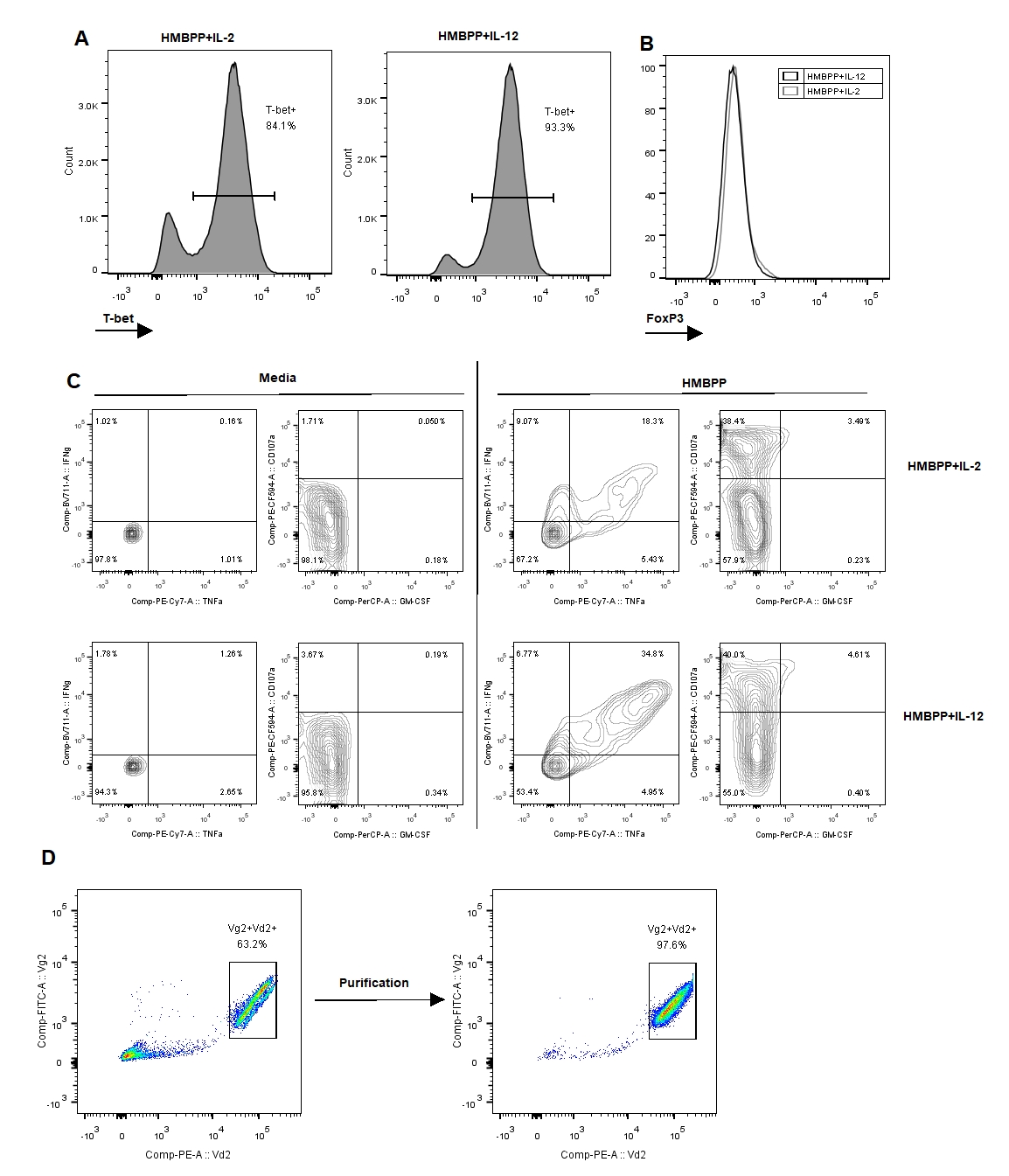


**C**


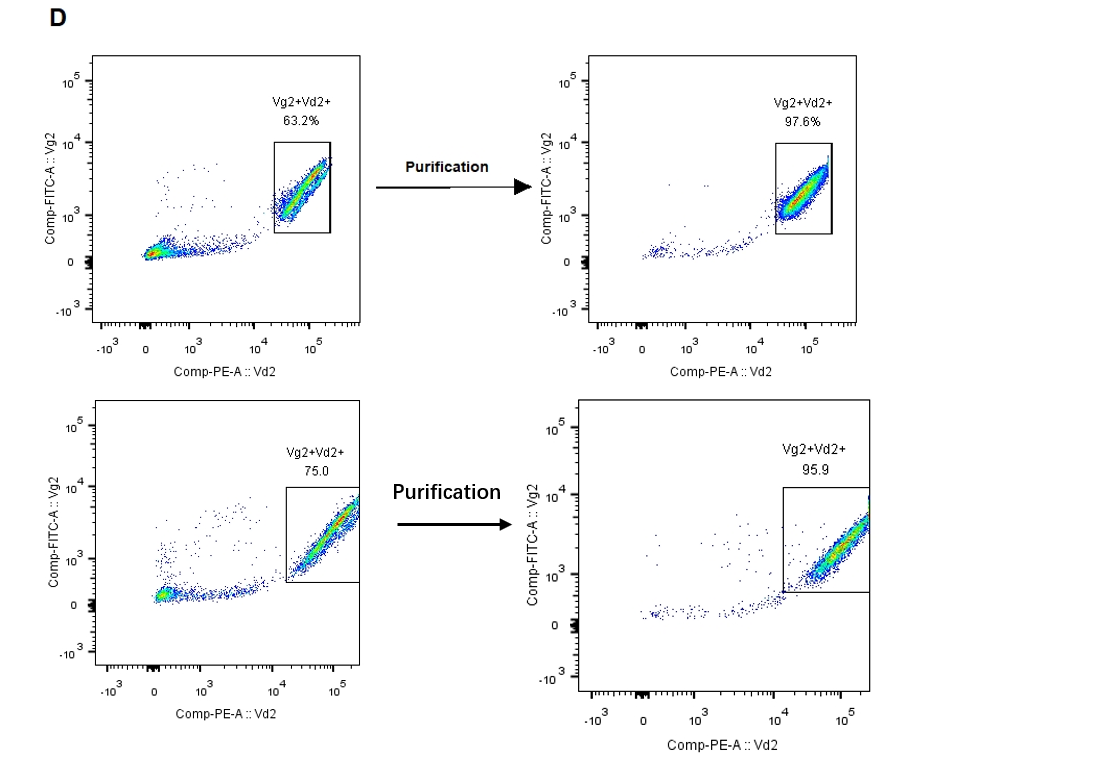


### Suppl Fig.4 Representative flow cytometry plots.

(A-B) Representative flow histograms of T-bet positive cells and MFI of FxoP3 in gated Vγ2Vδ2 T cells. (C) Purity of enriched Vγ2Vδ2 T cells for mycobacteria inhibition assay by MACS method. PBMCs were cultured under HMBPP+IL-12 for 7 days; then Vγ2Vδ2 T cells were purified by anti-Vδ2-PE and anti-PE-beads (see Methods in the text). Then, the purity of enriched Vγ2Vδ2 T cells was determined by surface staining. The purity of enriched population is 97 ± 2.2 % for HMBPP+IL-12 cocultures (upper panel) and 94 ± 2.06 % for HMBPP+IL-2 cocultures (lower panel) assessed by flow cytometry. Graph data shown as mean ± SD of 3 independent experiments pooled from 15 healthy controls.

**B**

**A**


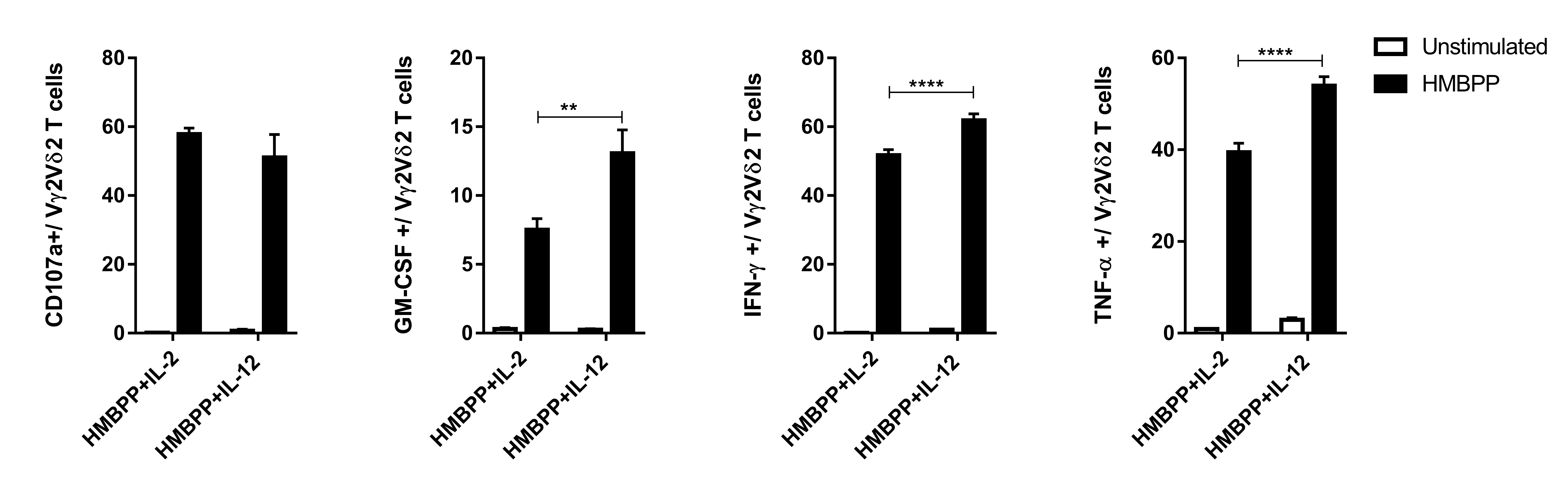


### Suppl Fig.5 HMBPP+IL-12 coculture differentiated higher frequencies of Vγ2Vδ2 T effector cells producing IFN-γ, TNF-α and GM-CSF than did the HMBPP+IL-2 co-treatment.

(A) Representative flow histograms showing coproduction of various cytokines by Vγ2Vδ2 T cells in response to HMBPP stimulation. The gating strategies of positive cytokines were based on the biology control, media group. PBMCs were cultured under HMBPP+IL-12 or HMBPP+IL-2 for 7 days, followed by HMBPP re-stimulation for 6 hours. Effector functions of expanded Vγ2Vδ2 T cells were determined by ICS measurement of intracellular cytokines. (B) Bar graph showing percentages of cytokine-producing Vγ2Vδ2 T cells in HMBPP+IL-12 or HMBPP+IL-2 cultures with or without HMBPP stimulation. Data are from 3 independent experiments pooled from 15 healthy controls. **** *p* < 0.0001, ** *p*<0.01, t test.
